# Whither the Rangeland?: Protection and Conversion in California's Rangeland Ecosystems

**DOI:** 10.1371/journal.pone.0103468

**Published:** 2014-08-20

**Authors:** D. Richard Cameron, Jaymee Marty, Robert F. Holland

**Affiliations:** 1 The Nature Conservancy, San Francisco, California, United States of America; 2 Geobotanical Phenomenology, Auburn, California, United States of America; University of California, Berkeley, United States of America

## Abstract

Land use change in rangeland ecosystems is pervasive throughout the western United States with widespread ecological, social and economic implications. In California, rangeland habitats have high biodiversity value, provide significant habitat connectivity and form the foundation for a number of ecosystem services. To comprehensively assess the conservation status of these habitats, we analyzed the extent and drivers of habitat loss and the degree of protection against future loss across a 13.5 M ha study area in California. We analyzed rangeland conversion between 1984 and 2008 using time series GIS data and classified resulting land uses with aerial imagery. In total, over 195,000 hectares of rangeland habitats were converted during this period. The majority of conversions were to residential and associated commercial development (49% of the area converted), but agricultural intensification was surprisingly extensive and diverse (40% across six categories). Voluntary enrollment in an agricultural tax incentive program provided widespread protection from residential and commercial conversions across 37% of the remaining rangeland habitat extent (7.5 M ha), though this program did not protect rangeland from conversion to more intensive agricultural uses. Additionally, 24% of the remaining rangeland was protected by private conservation organizations or public agencies through land or easement ownership while 38% had no protection status at all. By developing a spatial method to analyze the drivers of loss and patterns of protection, this study demonstrates a novel approach to prioritize conservation strategies and implementation locations to avert habitat conversion. We propose that this approach can be used in other ecosystem types, and can serve as a regional conservation baseline assessment to focus strategies to effect widespread, cost-effective conservation solutions.

## Introduction

The conversion of habitat from a natural state to developed human uses has fundamentally altered both the spatial configuration and function of ecosystems worldwide [1]. Compared to other ecosystems, the effects of habitat conversion on rangeland ecosystems have only received recent attention in the scientific literature [2]–[4]. This is despite the global vastness of rangelands, covering nearly one quarter to one third of the world's habitable land area [5], their high levels of biodiversity, and the large economic and social benefit provided by rangeland ecosystems [6], [7].

Rangeland habitats (grasslands, shrublands and woodlands) have also received less attention from conservation efforts than other major habitat types. The primary global biomes that support extensive rangelands– 1) Temperate grasslands, savannas and shrublands; and 2) Mediterranean forests, woodlands, and scrub– have experienced widespread conversion to anthropogenic land uses (45.8%, 41.4% converted respectively) and have the lowest proportion of legal conservation protection among all biomes (4.6%, 5.0% respectively) [8]. This habitat conversion is driven by population growth and associated residential and commercial development, as well as agricultural production. High levels of habitat loss are especially detrimental for biodiversity in Mediterranean regions of the world that have some of the highest population densities of all global biodiversity hotspots [9].

The economic and social benefits that grazed ecosystems provide vary by societal context. Many livestock-based economies operate in a subsistence mode, including 120 million pastoralists worldwide dependent on livestock as a primary food source [10]. In the coterminous western United States, rangelands are one of the most extensive land types covering over 163 million hectares, with approximately half of the land in non-federal ownership [11]. In addition to the subsistence and market-based economic values provided by rangelands, numerous ecosystem services are provided by grassland and woodland ecosystems as well, such as habitat for native pollinators [12], carbon sequestration [13]–[15] and water supply [16].

Rangelands contribute to global biodiversity by providing habitat for numerous species as well as critical habitat connectivity. Many iconic, wide-ranging species rely on the habitat connectivity provided by unfragmented rangelands to complete annual migrations in diverse regions such as eastern Africa, western North America and the Mongolian steppe [17]. In many rangeland ecosystems, native or livestock grazers can play a critical role in maintaining habitat diversity [18] or habitat for restricted endemic species [19], [20], yet impacts to ecosystems due to incompatible grazing are well-known, especially for riparian habitats in arid regions [21].

Despite being widespread with a rich historical legacy in the Western U.S., ranching is an economically marginal activity at small to medium scales of operation. For example, Wetzel et al. found that more than 70% of surveyed California ranchers make less than $10,000 profit annually [22]. In addition to the economic challenges of running smaller ranching operations and the demographic reality that the average age of ranchers is high, other factors have contributed to a declining population of ranchers, including impediments to intergenerational land-based wealth transfer, rise in rural residential amenity-based land purchases, and loss of critical industry infrastructure (such as processing facilities) [23]–[25]. These demographic and economic pressures increasingly lead to the dissolution of large land holdings into smaller parcels often resulting in the conversion of rangeland habitats into uses not compatible with ecosystem functioning [4], [26], [27].

The resulting fragmentation of rangeland habitat has many negative biological and social effects including loss of remote habitat, increase in human commensal species, severing of wildlife connectivity between core habitats, and an increasingly difficult social environment for ranching (e.g. new neighbors are affronted by certain aspects of a livestock operation) [2], [23], [28]. While the decision to break-up and sell a large ranch is made at the landowner or family level, the consequences become evident ecologically at the landscape scale with large areas converting to low density “exurbs” [3]. These diffuse development patterns represented the fastest growing land use in the country in recent decades [29].

To counteract these trends, various conservation strategies have been used, including voluntary, publicly-funded conservation and restoration incentive programs (such as the Grassland Reserve Program and the Wildlife Habitat Incentive Program), and acquisition of development rights through purchase of a conservation easement [30], [31]. These strategies can provide a range of conservation benefits including legal protection of land from conversion and improvement of habitat conditions for wildlife [31], but lack of transparency of terms and reporting makes a broad evaluation difficult [32]. Acquisition of fee title or certain rights to the land through a conservation easement requires the most upfront capital, but provides long-term assurance that the land will remain open habitat, if not actively grazed rangeland.

Conservation strategies such as reduction in property taxes for agricultural land uses through voluntary incentive programs can represent a significant boost to the economic viability of ranching [25]. One notable example of such a program was the California Land Conservation Act, commonly known as the Williamson Act (WA), that provided property tax reductions across six million hectares of the state as of 2009 [33]. Counties had been compensated by the State for lost property tax revenues ($35.1 M in 2009) but payments from the state to participating counties to compensate enrolled landowners stopped in 2009. Since then, the program has been implemented at the discretion of individual counties. Before 2009, to remove enrolled land from Williamson Act contracts, the landowner had to initiate a process of non-renewal which gradually restored the full tax burden over a nine year period. A primary reason that private landowners began the process of non-renewal was to plan for future subdivision and development, and as such it was a useful proxy for the threat of conversion to residential or commercial development. Other conservation tools such as habitat mitigation banks provide credits to offset development impacts to habitat for protected species and can provide conservation-friendly revenue streams for landowners. Future revenue from monetized ecosystem services including water banking and carbon sequestration is an area of widespread interest and active science and policy development throughout the western United States, yet substantial constraints remain [34], [35].

To determine the scope and drivers of rangeland habitat conversion, this study uses geographic data for development and agricultural land uses across a 13.5 M hectare portion of the California Floristic Province. California is an ideal place to look at rangeland habitat loss and conservation because of the globally significant biodiversity in the Mediterranean climate-influenced part of the state, high rate of population growth and progressive conservation policies and funding programs. While many aspects of California rangelands are unique relative to other western United States ranching landscapes, many of the social and economic drivers of land use change are similar to other regions, including demographic trends of ranchers and displacement of grazing for more profitable land uses. This study quantifies the amount of rangeland conversion that has occurred over 21 years in California and the level of protection afforded to the remaining rangelands. This information is critical to understanding the vulnerability of this ecosystem and associated social and economic benefits provided by ranching. This study also provides needed data for conservation planning efforts in California.

Using data from 1984 to 2008 on the extent of rangelands, we mapped areas that were converted from rangelands to other uses and, through aerial imagery interpretation, determined the resulting land use at the end of the time period. To assess the conservation status of remaining rangelands, we quantified the overlap between different short-term and permanent protective measures and rangeland ecosystem types. We present this approach as an attempt to convey the full spectrum of conservation status options, moving beyond traditional gap analyses that quantify the percentage of habitats that are in various categories of permanent legal protection [36], [37]. Such an adaptation of traditional analyses is critical for rangelands because of the importance of capturing the scope of voluntary programs that provide conservation benefits to landowners and society.

### Study area

**Figure 1:**
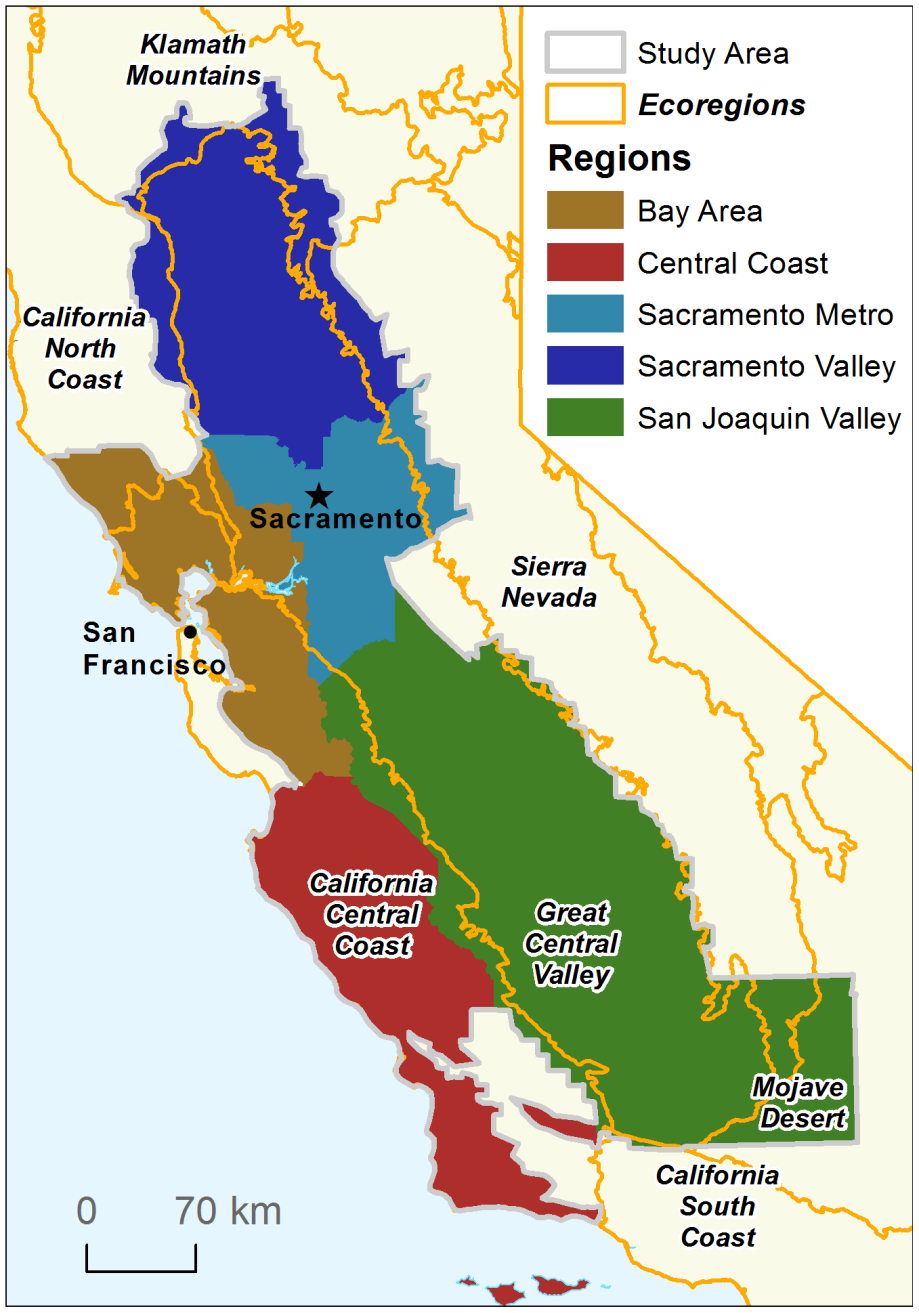
Study area. Extent of study area, ecoregions and reporting regions used for conversion analysis.

The study area encompasses the majority of the rangeland habitat within the California Floristic Province, including all of the Great Central Valley and California Central Coast ecoregions where data were available [38]. The study area also includes the full extent of Kern County which extends east into the southern part of the Sierra Nevada Mountains and western Mojave Desert because this portion of these ecoregions has similar habitats and land use to the southern Great Central Valley (Figure 1). The majority of the North and South Coast ecoregions were excluded from the analysis because they are not primarily in rangeland land cover. We defined our study area to align with the focus area of the California Rangeland Conservation Coalition (CRCC), a group of federal and state agencies, conservation organizations and ranchers working to develop collaborative solutions to make ranching economically viable and ecologically beneficial. As such, we focused on private rangelands and excluded the desert rangelands in the Northeast and Southeast part of California that are extensive, but have low productivity due to low rainfall and shorter growing seasons. All potential rangeland habitats in California cover 23.1 M hectares, of which approximately 60% was actually grazed in 2005 ([39].Four counties within the study area did not have data and were excluded from the analysis, Tuolumne and Calaveras in the Sierra Nevada foothills and San Mateo and Santa Cruz in the Central Coast ecoregion.

The rangeland habitats within the study area are dominated by Mediterranean annual grasslands and mixed hardwood woodlands, and are characterized by a strong latitudinal precipitation gradient. The highest average annual precipitation in the study area is near Redding, CA with 113 cm annually with the lowest in Kings County, near Taft with 14 cm annually. Coupled with the length of the growing season and differences in soil productivity, precipitation is a strong driver of forage productivity and quality, a major factor for the economic viability in ranching [40]. Forage productivity varies from 560 kg/ha to over 3900 kg/ha in areas closer to the coast [41].

## Methods

### Land use data

The California Farmlands Mapping and Monitoring Program (FMMP) was established in 1982 to provide consistent, objective information on agricultural land uses throughout California [42]. Eight land use categories are used to map general land use and evaluate the suitability of the land for irrigated agriculture (Prime, Statewide Importance, Unique, Locally Important) and to show the extent of grazing land, developed areas, water and other land uses. The grazing land category was used in this study to assess conversion patterns and is defined as “land on which the existing vegetation is suited to the grazing of livestock” (http://www.conservation.ca.gov/dlrp/fmmp/mccu/Pages/map_categories.aspx). The number of counties included in FMMP has increased over the years, now including over 95% of the privately held lands in California. The program uses soil survey GIS data, aerial imagery and field reconnaissance to map the land use categories described above every two years. Additional classes were added in 2002 for select San Joaquin Valley counties as part of the Rural Land Mapping project to further distinguish the Other Land category. We used the FMMP dataset because it represents a consistent land use change data source over a long time period at a relatively high spatial resolution (4 ha minimum mapping unit). Our study period matches the development of Geographic Information Systems (GIS) during which time the mapping protocol for the FMMP changed accordingly. Originally the mapping process used transparent aerial photos overlaid onto 100,000 scale paper topographic sheets. The resulting land use classification was then digitized for use in a GIS. Such changes introduce some error into any analysis of long-term data.

**Figure 2:**
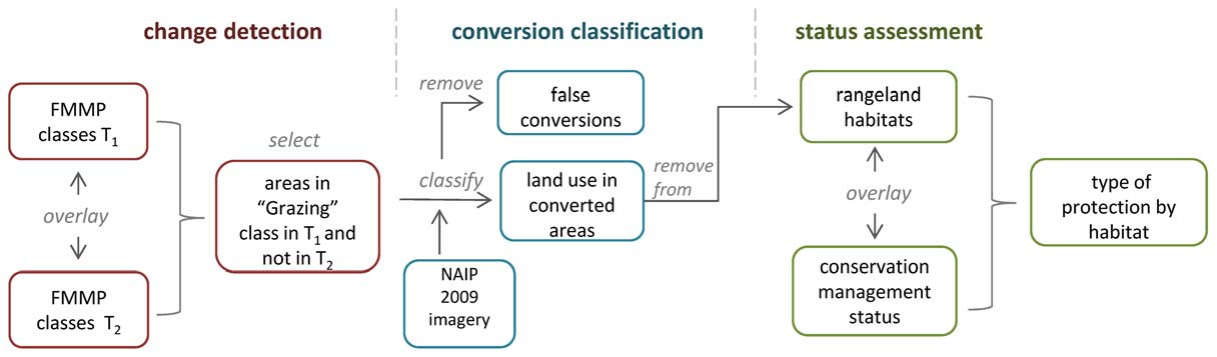
Sequence of core spatial analyses used in study. The three primary elements of the analysis are shown in sequence. The “change detection” step provided the full set of areas to classify in the “conversion classification.” Some lands where a change was detected but were not actually converted were removed from the analysis as false conversions. Converted lands were removed from the habitat data for the “status assessment”, which quantified the proportion of habitats under various types of protection. The year of the data used in FMMP overlays between time point 1 and time point 2 varied by survey area. Nineteen of the thirty-six survey areas spanned the period from 1984–2008, five spanned the period 1984–2006; and the remaining twelve covered various years with an average of seventeen years across them. The most protective conservation management status was assigned to land if multiple statuses existed, in order of precedence: fee ownership, easement, Williamson Act enrollment.

We assembled FMMP data for 33 counties throughout California for two points in time. The time period covered for each survey area (three counties were split into two survey areas) varied based on when FMMP mapping was initiated in a survey area and data availability at the time of the analysis. The beginning year was as early as 1984 and as late as 2000 and the end year was either 2006 or 2008, for a maximum range of 24 years. The range of years by survey area is shown in Table S1, and has a mean of 21 years across all survey areas. To map areas that were converted during this time, we overlaid the datasets from the beginning and end years and selected areas that were classified as Grazing Land in the first time period and another mapping category in the later period. We applied processing techniques to the composite data to reduce any errors resulting from small misalignments of the boundaries from the two years that may have resulted from the migration to a GIS-based workflow. Specifically, we forced any point along a line within 100 meters of a point from the other time period to be snapped together in a GIS. This resulted in simpler (less sinuous) polygon shapes and the removal of smaller and more linear features from the dataset. While this processing step was essential to be able to compare the data from the two time periods, it does limit analysis of the fine-scale fragmentation patterns resulting from conversion. Polygons smaller than four hectares were removed from the combined data set, as that was the minimum mapping unit for the FMMP land use data. A flow chart illustrating our analysis steps for the study is shown in Figure 2.

To assign a land use to the converted polygons, we used one-meter resolution, true color 2009 aerial imagery from the National Agriculture Imagery Program (NAIP) [43]. To distinguish the relative density of residential development, two categories were used: urban (housing density greater than 1 unit per .65 ha) and rural residential covering lower densities. During photo interpretation, a number of polygons were determined to be still in grazing land and not actual conversions and were a result of a change in classification. These were excluded from the analysis. Because the original FMMP classification does not identify crop types, we developed a new classification for this study that captures more variation in agricultural and developed land classes. We provide an assessment of the degree of overlap between these two classifications in Table S2.

To investigate the potential influence of the global financial crisis on land conversion rates, we assessed the rate of annual change from 2008 to 2010 for five counties that have different dominant land use types: Santa Barbara, Kern, Sacramento, Placer and Merced. We compared the rate of change for the years covered in this study and the rate for 2008–2010 to see if rates of conversion declined with economic activity.

### Private and public conservation management data

To assess the degree to which rangeland ecosystems are protected from conversion through legal mechanisms, we assembled land status data representing land held in fee or under conservation easement with a private conservation organization or public agency [44]. To estimate the conservation benefit of a voluntary, non-permanent conservation program in rangelands, we compiled spatial data on lands enrolled in the California Land Conservation (Williamson) Act [45] using the latest data available for each county, as recent as 2009. Yuba County was the only county in our study area that did not participate in the program. Williamson Act contracts were between a landowner and the county in which the enrolled land exists, and lasted a period of at least ten years after which point they needed to be renewed. We also included land covered by the “Super Williamson Act” contracts that lasted 20 years.

### Rangeland habitat data

We used the Multi-Source Land Cover data assembled by California Department of Forestry and Fire Protection [46] to represent the rangeland habitats of interest in the study area, classified using the California Wildlife Habitat Relationships (CWHR) standard [47]. Input data sources for this composite layer range from 1997–2002. We used a classification of rangelands used by the UC Davis Rangeland Watershed Lab as the primary list of habitats for this study (http://californiarangeland.ucdavis.edu/). We added the montane hardwood habitat type to the analysis as it is extensive within our study area and does experience some grazing depending on the location in the state, especially where California black oak (*Quercus kelloggii*) is dominant. We chose to use a habitat-based definition of rangeland extent and not the Grazing Land category from the FMMP data for the conservation status assessment because we wanted to quantify the variability of protective status across ecosystems that support domestic livestock. The diversity of ecological and social contexts for these ecosystems is high and differences in patterns of protection are useful to interpret the results across such a large study area.

We combined the data of rangeland habitats with the data for public land and privately protected areas, Williamson Act lands and converted rangeland areas within the GIS. To assess the conservation status of remaining rangelands we summarized the habitat distribution by five categories of land holdings: 1. private land, 2. private land enrolled in the Williamson Act, 3. private land not renewing Williamson Act contracts, 4. private land with a conservation easement, and 5. land held in fee title by a public agency or a non-governmental conservation organization. To facilitate reporting and interpretation, we divided the counties in the study area into distinct regions: the Bay Area, Sacramento Metro, Sacramento Valley, San Joaquin Valley, and Central Coast (Figure 1). Because geographic data on rangeland habitats do not exist before 1984, we were not able to determine which habitats were converted.

## Results

### Rangeland conversion

**Figure 3:**
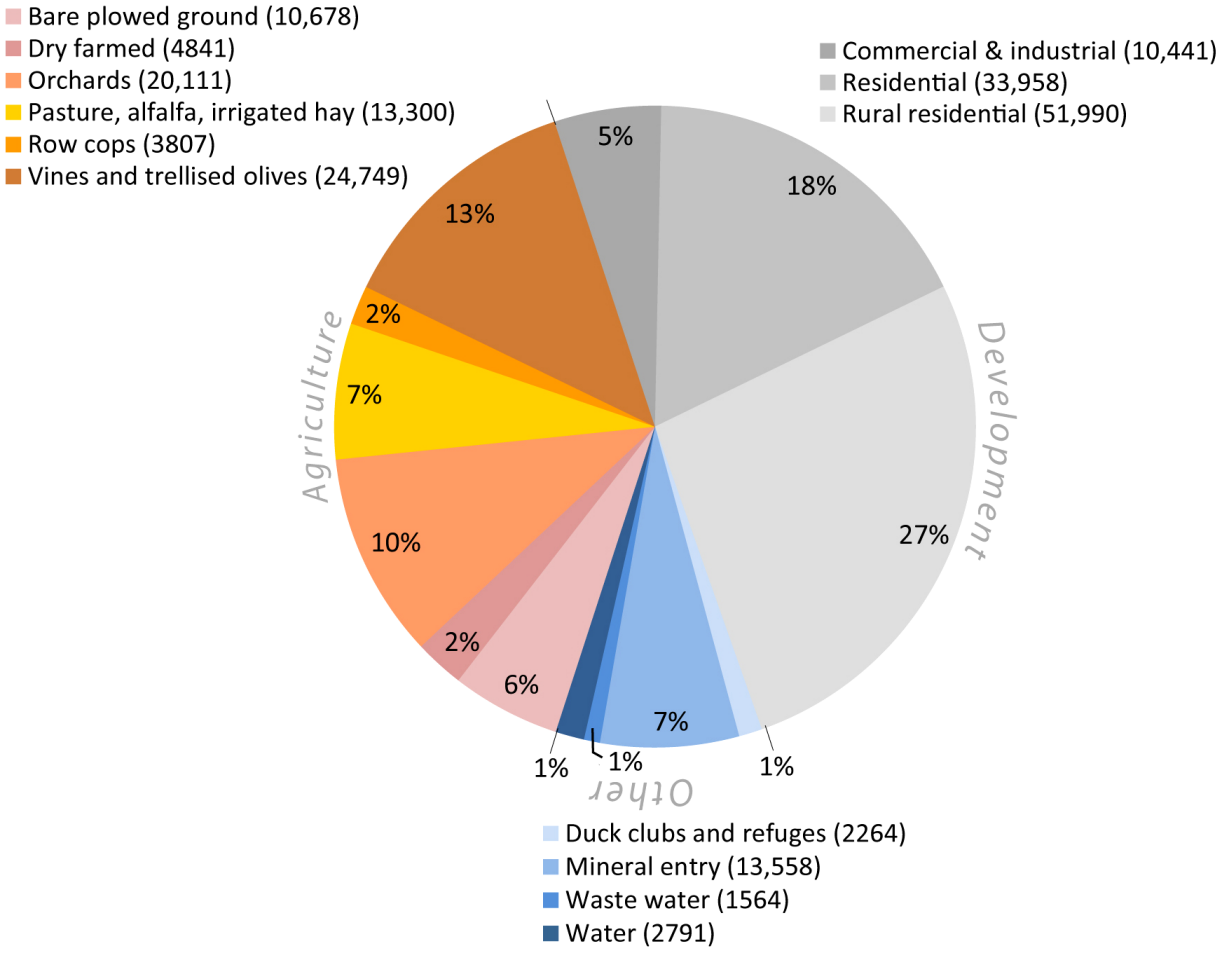
Area (Ha) of land cover types on land converted from rangelands during the study period. Converted areas were mapped using the California Department of Conservation's Farmland Mapping and Monitoring Program data (1984–2008) and classified using the National Agriculture Imagery Program imagery from 2009. The development categories (shown in gray) account for 49% of total area converted, agricultural conversions account for 40% and various other conversions account for about 10%.

Across the study area, 195,594 ha of rangelands were converted to other uses during the time period covered in the assessment. The most common conversions were to developed land classes (96,389 ha, 49% of the total, Figure 3) with the most extensive to rural residential followed by residential land. Loss of rangelands to more intensive agricultural land uses covered 78,793 ha (40% of total) with four land uses making up 87% of all conversions, including, in descending order, vines and trellised olives, orchards, pasture/alfalfa, and the transitional category of bare plowed ground. Other conversion types covered just over 20,000 ha and were dominated by mineral extraction (gravel, clay, oil/gas, tailings ponds) accounting for 66% of all area within this category.

**Figure 4:**
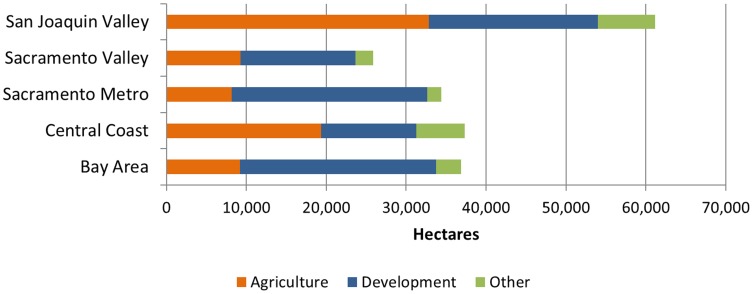
Regional conversion patterns by major type. The primary type of conversion for each region was variable, but can be explained partly by the existing land use in the region. For example, the more developed Sacramento Metro and Bay Area regions saw the majority of the converted land end up as residential and commercial development, while the majority of conversions in the San Joaquin Valley and Central Coast regions were to intensive agriculture classes. Conversion data are from an analysis of Farmland Mapping and Monitoring Program data, and land cover assigned using the National Agriculture Imagery Program imagery for 2009.

The San Joaquin Valley region experienced the largest amount of conversion (61,139 ha) with 54% of the converted area going to agricultural land uses (Figure 4). Conversion in the Bay Area and Sacramento Metro region was dominated by development, with the ratio of development to agricultural conversions at 2.7 and 3, respectively. In the Bay Area, the counties to the east of San Francisco Bay have the largest converted area collectively, but Sonoma County had the largest area converted of any of the Bay Area counties analyzed (Table S3).

**Figure 5:**
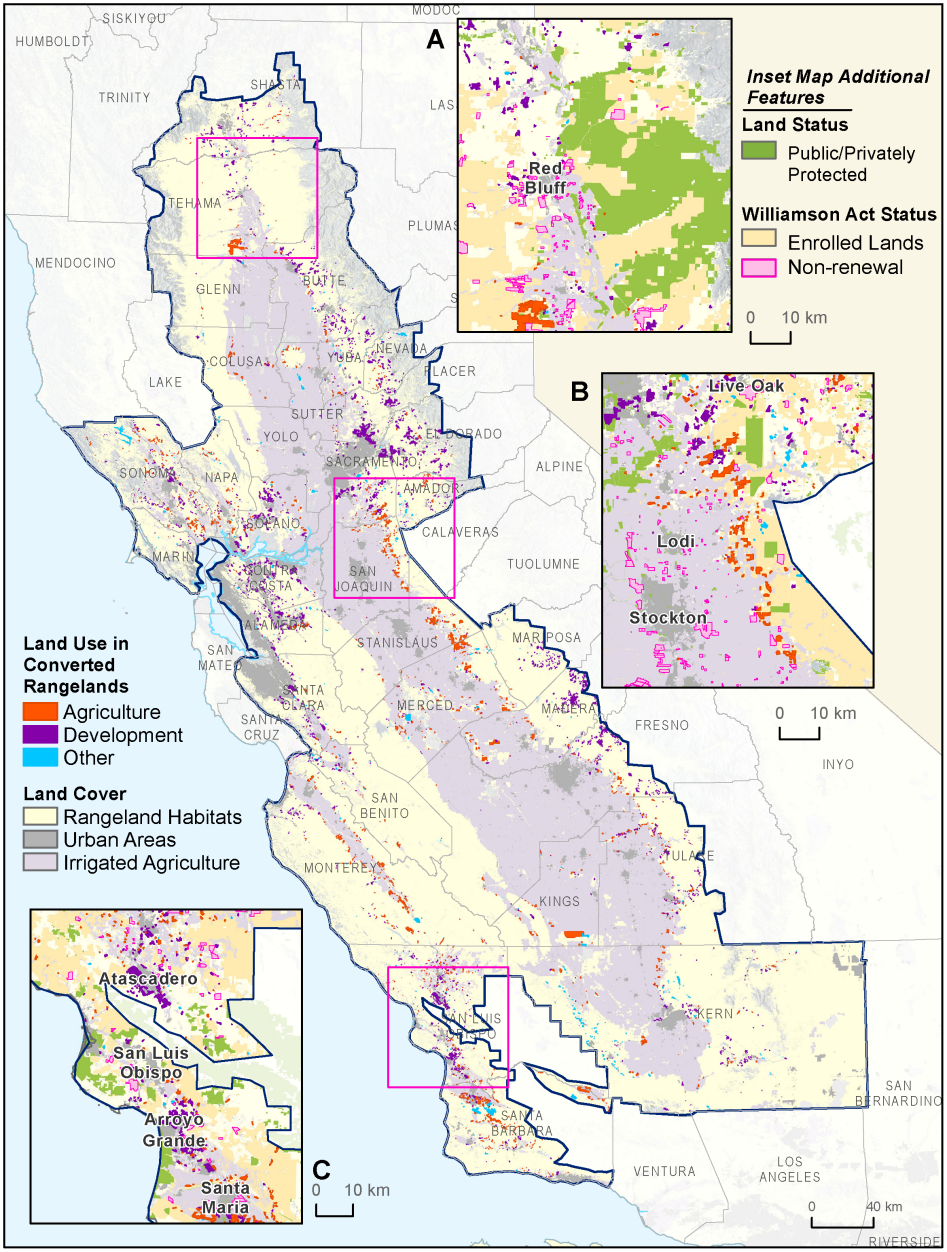
Conversion patterns by major type. The converted rangelands spanned a large geographic area. In some areas large protected area complexes were created where conversion was less widespread, possibly enabled by lower land prices (A). Agricultural conversions were more common adjacent to existing cropland (B). Conversions to developed land uses are dominated by new housing at lower densities at higher elevations in the Sierra Nevada foothills (Madera, Fresno, Butte counties) and at higher densities in the San Francisco Bay Area and near Sacramento. The areas in the Central Coast around Atascadero and Arroyo Grande experienced a large amount of conversion from a variety of sources (C).

The vast majority of the development in the Sacramento Metro region occurred in the grasslands and woodlands leading to the Sierra Nevada foothills east of Sacramento, with large conversions directly adjacent to the existing urbanized area (Figure 5). The Central Coast experienced slightly more conversion than the Bay Area or the Sacramento Region, from a diverse mix of conversion types. Agricultural conversions dominated Monterey County (primarily to vineyards) (Figure 5). In Santa Barbara County, large areas were converted to oil and gas extraction. San Luis Obispo County experienced dramatic residential growth around Arroyo Grande, Paso Robles and Atascadero totaling over 7269 ha in residential and rural residential classes (Figure 5B, Table S3).

The counties with more residential development had a slowdown in conversion rates from 2008–2010 with Sacramento and Placer declining 88% and 43%, respectively (Table 1). Santa Barbara County also experienced a drop of 40%, while Merced County's rate was 80% higher than historical suggesting that agricultural conversions did not slow in that county after the onset of the recession. Kern County also experienced a slight increase in the rate of conversions (18%) relative to the long-term average.

**Table 1:** Rate of conversion of rangeland for years up to 2008 and for 2008–2010 based on FMMP data.

County	Number of Years Included in Study (2008 and earlier)	Rate of conversion pre-2008 (Ha/Yr)	Rate of conversion 2008–2010 (Ha/Yr)
Santa Barbara	24	576	346
Kern	20	572	674
Merced	20	548	994
Sacramento	20	485	56
Placer	24	363	208

Counties with more agricultural land (Kern, Merced) experienced an increase in the rate of conversions compared to the long-term average, while Sacramento County experienced a decline in conversions likely due to the drop in demand for new housing.

### Conservation status

**Figure 6:**
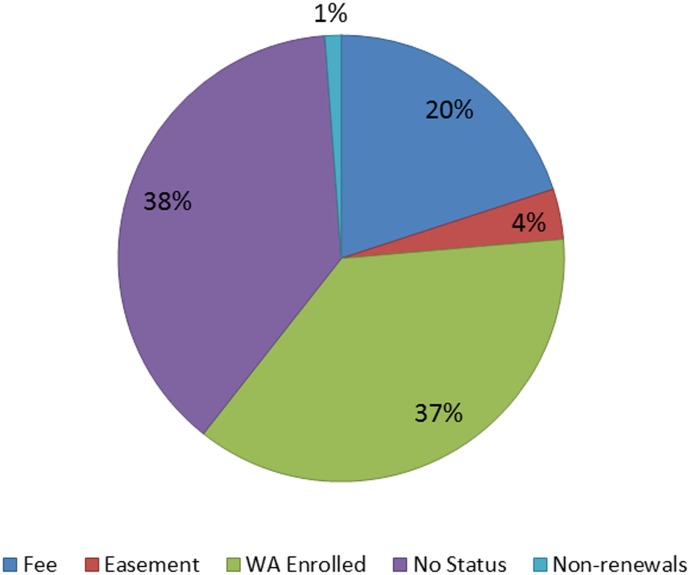
Conservation status of rangeland habitats. Over 60% of the total area in rangeland habitat have some protective status against various types of conversion with the majority provided by enrollment in the Williamson Act which prevented conversion to non-agricultural uses, but was defunded starting in 2009. This chart is for the portion of the study with rangeland habitats. WA = Williamson Act.

Of the remaining rangelands, 1.8M ha (24%) were protected against further conversion in fee title ownership or conservation easement held by a public agency or private conservation organization (Figure 6). In contrast, 2.8M ha (38%) of the rangeland area had no conservation status and was potentially subject to conversion to alternative land uses. Roughly the same amount of land (37%) was temporarily protected from conversion to development through enrollment in the Williamson Act.

The five most common habitat types made up 80% of the rangeland extent in the study area (Table 2), with a highly variable mix of permanent conservation status and WA enrollment. Lower elevation habitat in the Central Valley and Central Coast (annual grasslands and blue oak woodlands *(Quercus douglasii)*) were primarily conserved through enrollment in Williamson Act (49% and 46%, respectively), while higher elevation types in these regions (montane hardwood and mixed chaparral) were more commonly owned by a public entity, such as the U.S. Forest Service (23 and 44% fee, respectively). Desert scrub is most common in the southern San Joaquin Valley and western Mojave and had very little enrollment in the Williamson Act (1%) but substantial area in fee conservation status (42%). Other habitats that had a substantial percentage of area (>30%) enrolled in Williamson Act included coastal oak woodland, coastal scrub, blue oak-foothill pine and valley oak woodland.

**Table 2:** Area (Ha) by conservation status for California rangeland habitat types.

Habitat type	Total area	Fee Title	% Fee	Cons. Easement	% Easement	WA Enrolled	% WA	No status	% No status	WA Non-renewals	% Non- Renewal
Annual Grassland	3,186,845	355,034	11%	134,645	4%	1,586,176	50%	1,057,150	33%	53,840	1.68%
Blue Oak Woodland	1,073,109	120,142	11%	42,506	4%	503,270	47%	399,267	37%	7,923	0.74%
Montane Hardwood	676,434	157,316	23%	26,101	4%	144,555	21%	344,642	51%	3,819	0.56%
Mixed Chaparral	620,111	276,183	45%	17,283	3%	117,348	19%	207,128	33%	2,170	0.35%
Desert Scrub	445,750	190,521	43%	25	0%	5,936	1%	248,690	56%	578	0.13%
Coastal Oak Woodland	363,381	92,433	25%	19,971	5%	127,224	35%	114,981	32%	8,772	2.40%
Coastal Scrub	236,450	46,940	20%	9,762	4%	93,466	40%	81,460	34%	4,821	2.03%
Blue Oak-Foothill Pine	233,051	38,564	17%	7,262	3%	89,556	38%	95,968	41%	1,702	0.73%
Chamise-Redshank Chaparral	195,295	75,223	39%	6,749	3%	56,655	29%	55,751	29%	917	0.47%
Alkali Desert Scrub	173,106	19,532	11%	2,533	1%	10,114	6%	139,150	80%	1,777	1.03%
Pinyon-Juniper	71,857	51,727	72%		0%	5,948	8%	14,175	20%	7	0.01%
Valley Oak Woodland	42,150	3,933	9%	1,683	4%	14,300	34%	21,204	50%	1,030	2.43%
Montane Chaparral	40,077	18,615	46%	133	0%	4,196	10%	17,069	43%	64	0.16%
Sagebrush	29,510	19,742	67%	40	0%	1,846	6%	7,866	27%	15	0.05%
Valley Foothill Riparian	29,119	8,779	30%	2,378	8%	4,979	17%	12,711	44%	271	0.93%
Montane Riparian	26,431	4,427	17%	799	3%	7,188	27%	13,731	52%	285	1.08%
Juniper	15,505	4,850	31%		0%	3,067	20%	7,579	49%	9	0.06%
Bitterbrush	7,768	3,013	39%	38	0%	721	9%	3,996	51%		0.00%
Wet Meadow	5,818	1,966	34%	186	3%	1,192	20%	2,427	42%	47	0.81%
Desert Succulent Shrub	2,804	180	6%		0%	364	13%	2,259	81%		0.00%
Joshua Tree	1,704	848	50%		0%	45	3%	811	48%		0.00%
Desert Wash	1,606	1,111	69%	14	1%	23	1%	458	29%		0.00%
Perennial Grassland	1,409	387	27%	0	0%	213	15%	808	57%		0.00%
Total Study Area	7,479,287	1,491,466		272,106		2,778,384		2,849,281		88,050	

Data sources for fee and easement protected areas from GreenInfo Network. Williamson Act enrollment data from Department of Conservation, and rangeland habitat data from Calfire. WA = Williamson Act.

## Discussion

While rangeland conversion occurs at the ranch level, the decision to convert is driven by a complex and interacting network of economic and social factors including the incentives and disincentives provided by land use policy. The ecological and economic threats to rangelands are numerous (invasive species, climate change, lack of profitability), but the conversion to development or intensive agriculture is often the most permanent. Conversion to more intensive crop types, such as orchards and vineyards, often eliminates critical endangered species habitat (e.g. vernal pools) and fundamentally alters the structure and function of these lands for wildlife use [48]. In a similar study, the extent of vernal pools in California rangelands decreased by 13% from 1976 to 2005 [49]. Agricultural conversion requiring the irreversible mechanical ripping of impervious soil layers characteristic of vernal pools accounted for over 80 percent of this habitat loss.

While conversion of rangelands to plowed, irrigated crops represents a loss of terrestrial habitat, it also impacts aquatic ecosystems through surface or groundwater use [50]. Conversion of oak savanna to vineyards has been shown to represent a net increase in groundwater stress, especially in the dry summer months [51]. The conversion of rangeland to irrigated crops puts excessive demands on already depleted groundwater resources, particularly during drought conditions where managed surface water resources are restricted and groundwater is the only source of agricultural water [52]. These secondary effects of conversion need to be considered when conducting land use planning for rural parts of the state, particularly in the context of projected changes due to climate change. Compared to annual crops, nuts and vines constitute a “hardening” of water use in that the crops will fail without water whereas fields of annual crops can be fallowed to conserve water resources.

Changes in consumption patterns in wine, almonds, olives and other orchard crops may be driving the expansion of these crop types in rangeland ecosystems. Wine grape acreage increased in the state during the time period covered in this study by 56% [53] with the contribution of the northern Central Valley outpacing the increase in more traditional wine regions such as Napa and Sonoma counties, due to the region's role in producing varietals whose popularity has grown in recent decades, such as merlot, chardonnay and cabernet sauvignon [54]. Increases in international consumption of almonds and olives may be contributing to the expansion of these crops in the region. Almonds are primarily grown for international export (67% of production exported in 2010) with the European Union, China and India being the largest importers. The harvested area for almonds in California increased by 78% (over 120,000 ha) from 1984 to 2008 [55].

While this analysis classified the land use in 2009 in converted areas, we cannot necessarily assume that the 2009 land use was the original driver for conversion, as the original conversion may have occurred 20 or more years earlier. Other studies have shown that land use change in California is very dynamic with many conversions between grasslands/shrublands ecosystems and agriculture due to fallowing of agricultural land and the impact of longer drought cycles [27], [56], [57]. Additionally, development conversions are often preceded by agricultural conversions [58]. Given that, we assume that some of the areas that were classified as residential development in 2009 were initially converted from rangeland to agricultural types, though likely to annual crops rather than perennial crops (e.g. orchards, vineyards). Interactions between conversion types have been observed elsewhere [59] and have been proposed to explain some rangeland conversion in California [27]. The theory holds that when prime farmland is converted to residential and commercial development at the edge of cities, it causes a displacement of agricultural land uses to areas with more marginal soils, such as rangelands [59]. It is likely that as more marginal soils are exploited, the fertilizer inputs would increase to maintain productivity and that the sandier, well-drained soils would require more irrigation than a similar crop in better soils [50]. Thus, the cumulative effects of development of prime farmland need to be a factor in future land use planning in the Central Valley, and other rapidly growing regions with extensive farmland.

Post-recession rates of change revealed variability in the response to the economic downturn in 2008. Yet, further investigation of the response of different land uses to catastrophic events, both economic and natural, is needed to help interpret this limited comparison. Higher temporal resolution studies of economic and climatic drivers of land use change can yield helpful information for planners, and can provide a complementary perspective to this long-term assessment of change [57].

Our results show that over a third of the rangeland area has no protective legal status, suggesting that some of these areas may be at risk to future habitat loss. Yet the drivers on land use change are spatially variable, often corresponding to proximity to existing development and infrastructure, and unprotected land is not necessarily at risk of development within the scale of a few decades. There may be local protective ordinances or zoning that benefit ranching landowners in these areas, but such an assessment is beyond the scope of this study. Permanent habitat protection is relatively low across all rangeland types (24%) with a strong bias toward more montane and desert habitat types. Some of these areas may be protected from development but not grazed, such as State Parks, because of other resource priorities (e.g. recreation or water quality) and as such represent an area of trade-off between these values and livestock production, and any ecological benefits associated with grazing.

Conservation easements are currently an underutilized strategy for rangeland protection, with only small proportions of annual grasslands (4%) and blue oak woodlands (3%) covered by these agreements. While a lack of standardized data collection on easement locations likely make this an underestimate, easements still cover a very small percentage of foothill habitats. As it is a voluntary solution that can provide conservation benefits to landowners and society while providing an infusion of money into ranching operations, conservation easements should be prioritized in public and private funding for rangeland conservation [60].The foothills of Mt. Lassen in the northern Central Valley is a region where extensive rangeland easements have helped limit the expansion of development from the city of Red Bluff while creating a connected network of protected lands stretching from valley floor to subalpine habitats (Figure 5A).

The results of this study can be used to prioritize general areas to implement conservation strategies such as purchasing easements or focusing ecological restoration efforts. The data from this study can be integrated into habitat connectivity assessments for example, to quantify the level of fragmentation from conversion in potential linkages between existing core habitat. While other factors need to be considered for site-specific project implementation, broad priority areas can be identified by analyzing patterns of conversion and types of land protection such as presented here. Similar analyses can be used to identify areas where land use policies that maintain extensive agriculture such as livestock grazing may be needed to prevent loss of rangelands, if that is an objective of the local jurisdiction.

The Williamson Act had benefited landowners in California across a large area covered by rangelands for 44 years, especially for annual grasslands and blue oak woodlands, the two most extensive habitat types (49% and 46% respectively). Other studies have highlighted the importance of these property tax benefits for maintaining ranching operations [22], [25], but it is not a panacea for rangeland ecosystem protection. An analysis of the conservation status and conversion datasets used in this study showed that 53% of the areas converted to vineyards were enrolled in Williamson Act in 2009. As such, it is clear that the Act did not prevent rangeland loss to more intensive agricultural land uses. Refinements to a restored Williamson Act, or subsequent legislation, that protect the broader array of public benefits including wildlife habitat, may be needed to prevent the agricultural intensification of rangelands. Yet, given the extensive area of enrolled lands, the benefits of the law for habitat protection were immense and restoring the Williamson Act should be a statewide land use policy priority. It is highly likely that increased development would result if subvention payments from the State or counties to landowners disappear altogether [22]. In fact, we see some evidence of that occurring in the most recent report on Williamson Act enrollment [61]. There was a decline of 14.5% in annual enrollment in 2010–2011 and a sharp increase in non-renewals in 2011 of 69,000 hectares compared to the annual average of non-renewals of 30,000 for the previous decade.

While we have taken one step toward a full spectrum conservation status analysis by including enrollment in the Williamson Act and conservation easements, the status assessment highlights the need to integrate multiple types of data to develop a comprehensive picture of conversion and conservation. Because conservation in private lands relies on a diverse array of voluntary, incentive-based programs that provide direct payments, tax relief or regulatory streamlining in exchange for beneficial management, often at the scale of individual ranches, we need better systems to track and account for the cumulative benefit of these programs. Integrating such programmatic data with change detection analyses such as this one can help develop a regional conservation accounting system that can be used to generate an ecological “P&L” statement, one of *protection* and *loss*. Inventorying and mapping the broad range of beneficial restoration actions that are publicly funded to promote agricultural best management practices or habitat restoration will provide a more nuanced and complete picture of what conservation benefits are realized by what interests [62].

Ultimately, the public pays to replace the goods and services lost when natural habitats are converted, such as pollination of crops and development of water storage and delivery systems [12], [52]. In addition, when conversions take place near existing protected land, the ecological connectivity value of that land is eroded due to habitat fragmentation. If the protected land was acquired with public funds, then society pays twice for that investment, once for the initial purchase and again to replace the ecosystem service benefits that are lost by adjacent habitat loss. Developing alternative markets to compensate landowners for the additive ecosystem benefit of beneficial management decisions (e.g. increased soil carbon storage as an emissions offset) may ultimately provide critical revenue to improve the economic viability of ranching operations [15], [63]

## Conclusion

Creating and implementing solutions to the problem of rangeland habitat loss and associated impacts will require an understanding of the economic and social drivers that lead to the breakup of larger land holdings, particularly those drivers related to global agricultural commodities. Solving such challenges requires cross-disciplinary collaboration to create strategies that address the various social contexts for ranching. The numerous ecological and social benefits provided by rangeland ecosystems in the Western United States can only be sustained if economic incentives are promoted to maintain ecologically sustainable grazing operations across large land ownerships. Accounting for the full economic, ecological and social costs of rangeland conversion would provide decision-makers with a greater ability to weigh trade-offs associated with land use change.

## Supporting Information

Table S1 **Time range by county or survey area for FMMP data.** The average range of all survey area is 21 years.
(DOCX)

Table S2 **Classification agreement between the Farmland Mapping and Monitoring Program (FMMP) categories and the classification used in this study.** B- Bare Plowed Ground; CI- Commercial and Industrial; DB- Dirt Bikes; DF- Dry farmed; DCR- Duck clubs and refuges; ME- Mineral entry: gravel, clay, oil fields, tailings; N- Nurseries; O- Orchards; P- Pasture, alfalfa, irrigated hay; R- Residential; Ri- Rice; RC- Row crops; RR- Rural residential; U- Unknown; V- Vines and trellised olives; WW- Wastewater; W- Water.
(DOCX)

Table S3 **Conversion types by counties.** Area in hectares.
(DOCX)

## References

[1] Vitousek PM (1994) Beyond global warming: ecology and global change. Ecology 75: 1861–1876.

[2] Maestas JD, Knight RL, Gilgert WC (2003) Biodiversity across a Rural Land-Use Gradient. Conservation Biology 17: 1425–1434.

[3] Theobald DM (2005) Landscape Patterns of Exurban Growth in the USA from 1980 to 2020. Ecology and Society 10.

[4] Hansen AJ, Knight RL, Marzluff JM, Powell S, Brown K, et al (2005) Effects of Exurban Development on Biodiversity: Patterns,. Mechanisms and Research Needs Ecological Applications 15: 1893–1905.

[5] Blench R, Sommer F (1999) Understanding Rangeland Biodiversity. London: Overseas Development Institute.

[6] Knight RL (2007) Ranchers as a Keystone Species in a West That Works. Rangelands 29: 4–9.

[7] FAO (2011) World Livestock 2011: Livestock in Food Security. Rome

[8] Hoekstra JM, Boucher TM, Ricketts TH, Roberts C (2005) Confronting a biome crisis: global disparities of habitat loss and protection. Ecology Letters 8: 23–29.

[9] Cincotta RP, Wisnewski J, Engelman R (2000) Human population in the biodiversity hotspots. Nature 404: 990–992. 10801126 10.1038/35010105

[10] Rass N (2006) Policies and Strategies to Address the Vulnerability of Pastoralists in Sub-Saharan Africa. Rome: FAO.

[11] USFS (1989) An Analysis of the Land Base Situation in the United States 1989–2040. U.S. Forest Service. 77 p.

[12] Chaplin-Kramer R, Tuxen-Bettman K, Kremen C (2011) Value of Wildland Habitat for Supplying Pollination Services to Californian Agriculture. Rangelands 33: 33–41.

[13] Christensen GA, Campbell SJ, Fried JS (2008) California Forest Resources 2001–2005. USDA Forest Service Pacific Northwest Research Station. 183 p.

[14] Silver WL, Ryals R, Eviner V (2010) Soil Carbon Pools in California's Annual Grassland Ecosystems. Rangeland Ecology & Management 63: 128–136.

[15] Derner JD, Schuman GE (2007) Carbon sequestration and rangelands: A synthesis of land management and precipitation effects. Journal of Soil and Water Conservation 62: 77–85.

[16] Havstad KM, Peters DPC, Skaggs R, Brown J, Bestelmeyer B, et al (2007) Ecological services to and from rangelands of the United States. Ecological Economics 64: 261–268.

[17] Harris G, Thirgood S, Hopcraft JGC, Cromsigt J, Berger J (2009) Global decline in aggregated migrations of large terrestrial mammals. Endangered Species Research 7: 55–76.

[18] Olff H, Ritchie ME (1998) Effects of herbivores on grassland plant diversity. Trends in Ecology & Evolution 13: 261–265. 21238294 10.1016/s0169-5347(98)01364-0

[19] Murphy DD, Weiss SB (1988) Ecological studies and the conservation of the bay checkerspot butterfly, *Euphydryas editha bayensis* . Biological Conservation 46: 183–200.

[20] Marty JT (2005) Effects of cattle grazing on diversity in ephemeral wetlands. Conservation Biology 19: 1626–1632.

[21] Belsky AJ, Matzke A, Uselman S (1999) Survey of livestock influences on stream and riparian ecosystems in the western United States. Journal of Soil and Water Conservation 54: 419–431.

[22] Wetzel WC, Lacher IL, Swezey DS, Moffitt SE, Manning DT (2012) Analysis reveals potential rangeland impacts if Williamson Act eliminated. California Agriculture 66.

[23] Liffmann RH, Huntsinger L, Forero LC (2000) To ranch or not to ranch: home on the urban range? Journal of Range Management 53: 362–370.

[24] Gosnell H, Haggerty J, Travis W (2006) Ranchland Ownership Change in the Greater Yellowstone Ecosystem, 1990–2001: Implications for Conservation. Society and Natural Resources 19: 743–758.

[25] Brunson MW, Huntsinger L (2008) Ranching As A Conservation Strategy: Can Old Ranchers Save The New West? Rangeland Ecology & Management 61: 137–147.

[26] Mitchell JE, Knight RL, Camp RJ (2002) Landscape Attributes Of Subdivided Ranches. Rangelands 24: 3–9.

[27] Sleeter B, Wilson T, Soulard C, Liu J (2011) Estimation of late twentieth century land-cover change in California. Environmental Monitoring and Assessment 173: 251–266. 20217217 10.1007/s10661-010-1385-8

[28] Odell EA, Knight RL (2001) Songbird and Medium-Sized Mammal Communities Associated with Exurban Development in Pitkin County, Colorado. Conservation Biology 15: 1143–1150.

[29] Brown DG, Johnson KM, Loveland TR, Theobald DM (2005) Rural Land-Use Trends in the Conterminous United States, 1950–2000. Ecological Applications 15: 1851–1863.

[30] Sokolow AD, Lemp C (2002) Agricultural easement programs. Saving agriculture or saving the environment? California Agriculture 56: 9–14.

[31] Rissman AR, Lozier L, Comendant T, Kareiva P, Kiesecker JM, et al (2007) Conservation Easements: Biodiversity Protection and Private Use. Conservation Biology 21: 709–718. 17531049 10.1111/j.1523-1739.2007.00660.x

[32] Merenlender AM, Huntsinger L, Guthey G, Fairfax SK (2004) Land Trusts and Conservation Easements: Who Is Conserving What for Whom? Conservation Biology 18: 65–76.

[33] CDOC (2010) California Land Conservation (Williamson) Act Status Report. Sacramento. CA: CA Department of Conservation

[34] Diaz D, Rashford B, De Gryze S, Zakreski S, Dell R, et al.. (2012) Evaluation of Avoided Grassland Conversion and Cropland Conversion to Grassland as Potential Carbon Offset Project Types. Portland, Oregon: The Climate Trust.

[35] Booker K, Huntsinger L, Bartolome JW, Sayre NF, Stewart W (2013) What can ecological science tell us about opportunities for carbon sequestration on arid rangelands in the United States? Global Environmental Change 23: 240–251.

[36] Davis FW, Stoms DM, Hollander AD, Thomas KA, Stine PA, et al (1998) The California Gap Analysis Project–Final Report. Santa Barbara UC Santa Barbara

[37] Scott JM, Davis F, Csuti B, Noss R, Butterfield B, et al (1993) Gap Analysis: A geographic approach to protection of biodiversity. Wildlife Monographs 123: 1–41.

[38] TNC (2006) California Ecoregions. San Francisco, CA: The Nature Conservancy.

[39] Calfire (2010) California's Forests and Rangelands: 2010 Assessment. Sacramento, CA: CA Department of Forestry and Fire Protection

[40] George M, Bartolome J, McDougald N, Connor M, Vaughn C, et al. (2001) Annual Range Forage Production. University of California, Division of Agriculture and Natural Resources. 9 p.

[41] NRCS (2012) Soil Survey Washington, D.C. : USDA, Natural Resources Conservation Service.

[42] CDOC (2008) Farmlands Mapping and Monitoring Program. Sacramento, CA: CA Dept. of Conservation.

[43] FSA (2009) National Agriculture Imagery Program. Washington, D.C.: USDA Farm Services Agency.

[44] GIN (2011) California Protected Areas Database. San Francisco, CA: GreenInfo Network.

[45] CDOC (2010) Williamson Act land status. Sacramento, CA: CA Dept. of Conservation.

[46] Calfire (2006) Multisource Land Cover dataset, fveg06. Calfire, Sacramento, CA.

[47] Mayer KE, Laudenslayer WF (1988) A Guide to the Wildlife Habitats of California. Sacramento, CA.

[48] Kucera TE, Barrett RH (1995) Displaced by agriculture, urban growth: California wildlife faces uncertain future. California Agriculture 49: 23–27.

[49] AECOM (2009) Loss of Central Valley Vernal Pools: Land Conversion, Mitigation Requirements, and Preserve Effectiveness. Sacramento, CA. 15 p.

[50] Charbonneau R, Kondolf GM (1993) Land use change in California, USA: Nonpoint source water quality impacts. Environmental Management 17: 453–460.

[51] Grismer ME, Asato C (2012) Converting oak woodland or savanna to vineyards may stress groundwater supply in summer. California Agriculture 66: 144–152.

[52] Famiglietti JS, Lo M, Ho SL, Bethune J, Anderson KJ, et al (2011) Satellites measure recent rates of groundwater depletion in California's Central Valley. Geophysical Research Letters 38: L03403.

[53] USDA (2011) California Wine Grapes 1920–2010. Washington, D.C.

[54] Volpe RJ, Green R, Heien D, Howitt R (2010) Wine-grape production trends reflect evolving consumer demand over 30 years. California Agriculture 64: 42–46.

[55] NASS (2011) California Historic Commodity Data: Almonds 1909–2010. U.S. Department of Agriculture.

[56] Sleeter BM (2008) Late 20th century land change in the Central California Valley Ecoregion. The California Geographer 48: 27–60.

[57] Soulard CE, Wilson TS (2013) Recent land-use/land-cover change in the Central California Valley. Journal of Land Use Science 1–22.

[58] Huston M (2005) The Three Phases of Land-use Change: Implications for Biodiversity. Ecological Applications 15: 1864–1878.

[59] Greene RP, Stager J (2001) Rangeland to cropland conversions as replacement land for prime farmland lost to urban development. The Social Science Journal 38: 543–555.

[60] Rissman AR, Reiner R, Merenlender AM (2007) Monitoring Natural Resources on Rangeland Conservation Easements. Rangelands 29: 21–26.

[61] CDOC (2013) The California Land Conservation Act 2012 Status Report. Sacramento. CA: CA Department of Conservation

[62] Huntsinger L, Johnson M, Stafford M, Fried J (2010) Hardwood Rangeland landowners in California from 1985 to 2004: Production, Ecosystem Services, and Permanence. Rangeland Ecology & Management 63: 324–334.

[63] Gosnell H, Robinson-Maness N, Charnley S (2011) Engaging Ranchers in Market-Based Approaches to Climate Change Mitigation: Opportunities, Challenges, and Policy Implications. Rangelands 33: 20–24.

