# Supplementary material for: Whither the Rangeland?: Protection and Conversion in California's Rangeland Ecosystems
Source: PLoS One. 2014 Aug 20;9(8):e103468. doi: 10.1371/journal.pone.0103468 (PMC4139198; doi:10.1371/journal.pone.0103468)
Supplement: Table S1 — Time range by county or survey area for FMMP data. The average range of all survey area is 21 years. (DOCX) [file pone.0103468.s001.docx]

| County Name | Beginning Year | End Year | Number of Years |
| --- | --- | --- | --- |
| Alameda | 1984 | 2008 | 24 |
| Amador | 1984 | 2008 | 24 |
| Butte | 1988 | 2008 | 20 |
| Colusa | 1986 | 2006 | 20 |
| Contra Costa | 1984 | 2008 | 24 |
| El Dorado | 1984 | 2008 | 24 |
| Fresno-East | 1984 | 2008 | 24 |
| Fresno-West | 2000 | 2008 | 8 |
| Glenn | 1984 | 2008 | 24 |
| Kern | 1988 | 2008 | 20 |
| Kings | 1984 | 2006 | 22 |
| Madera | 1984 | 2008 | 24 |
| Marin | 1984 | 2008 | 24 |
| Mariposa | 1984 | 2008 | 24 |
| Merced-East | 1984 | 2008 | 24 |
| Merced-West | 1992 | 2008 | 16 |
| Monterey | 1984 | 2008 | 24 |
| Napa | 1984 | 2008 | 24 |
| Nevada | 1984 | 2006 | 22 |
| Placer | 1984 | 2008 | 24 |
| Sacramento | 1988 | 2008 | 20 |
| San Benito | 1984 | 2008 | 24 |
| San Joaquin | 1990 | 2006 | 16 |
| San Luis Obispo | 1984 | 2006 | 22 |
| Santa Barbara | 1984 | 2008 | 24 |
| Santa Clara | 1984 | 2008 | 24 |
| Shasta | 1984 | 2008 | 24 |
| Solano | 1984 | 2008 | 24 |
| Sonoma | 1984 | 2006 | 22 |
| Stanislaus- East | 1998 | 2008 | 10 |
| Stanislaus- West | 2000 | 2008 | 8 |
| Sutter | 1988 | 2006 | 18 |
| Tehama | 1984 | 2006 | 22 |
| Tulare | 1986 | 2008 | 22 |
| Yolo | 1984 | 2008 | 24 |
| Yuba | 1986 | 2008 | 22 |
| Average |  |  | 21 |
