# Supplementary material for: Whither the Rangeland?: Protection and Conversion in California's Rangeland Ecosystems
Source: PLoS One. 2014 Aug 20;9(8):e103468. doi: 10.1371/journal.pone.0103468 (PMC4139198; doi:10.1371/journal.pone.0103468)
Supplement: Supplementary file 2 [file pone.0103468.s002.docx]

FMMP Categories	B	CI	DB	DF	DCR	ME	N	O	P	R	Ri	RC	RR	U	V	WW	W	Total Area (Ha)
Confined Animal Agriculture	0%	88%	0%	0%	0%	1%	0%	0%	2%	0%	0%	0%	0%	0%	2%	7%	0%	1,017
Developed	1%	13%	0%	0%	0%	3%	0%	0%	0%	76%	0%	0%	3%	0%	0%	3%	0%	39,568
Farmland of Local Importance	16%	2%	0%	18%	0%	0%	0%	4%	22%	2%	0%	1%	28%	0%	8%	0%	0%	15,562
Prime or Statewide Soil and not cultivated	9%	26%	0%	15%	0%	0%	0%	0%	0%	8%	0%	0%	30%	0%	13%	0%	0%	401
Nonagricultural or Natural Vegetation	1%	1%	0%	2%	44%	3%	0%	0%	6%	0%	0%	0%	40%	0%	0%	0%	2%	3,000
Prime Farmland	8%	0%	0%	7%	0%	0%	0%	26%	22%	1%	2%	5%	1%	0%	28%	0%	0%	12,542
Rural Residential Land	0%	1%	0%	0%	0%	0%	0%	0%	0%	3%	0%	0%	96%	0%	0%	0%	0%	11,854
Farmland of Statewide Importance	8%	0%	0%	4%	0%	0%	0%	18%	28%	0%	0%	15%	0%	0%	26%	0%	0%	9,959
Semi-Agricultural and Rural Commercial Land	0%	76%	3%	0%	0%	1%	0%	3%	0%	0%	0%	0%	16%	0%	1%	0%	0%	447
Unique Farmland	4%	0%	0%	1%	0%	0%	1%	36%	9%	0%	1%	4%	0%	0%	43%	0%	0%	39,736
Vacant or Disturbed Land	49%	3%	0%	0%	0%	45%	0%	0%	0%	0%	0%	0%	1%	0%	0%	0%	0%	8,864
Water	0%	0%	2%	0%	0%	0%	0%	0%	0%	0%	0%	0%	0%	0%	2%	0%	97%	2,100
Other	1%	6%	0%	0%	2%	16%	0%	0%	1%	6%	0%	0%	66%	0%	1%	0%	1%	50,546
