# Supplementary material for: Whither the Rangeland?: Protection and Conversion in California's Rangeland Ecosystems
Source: PLoS One. 2014 Aug 20;9(8):e103468. doi: 10.1371/journal.pone.0103468 (PMC4139198; doi:10.1371/journal.pone.0103468)
Supplement: Supplementary file 3 [file pone.0103468.s003.docx]

County	Rural residential	Residential	Vines and trellised olives	Orchards	Mineral entry	Pasture, alfalfa, irrigated hay	Bare plowed ground	Commercial & industrial	Dry farmed	Row cops	Water	Duck clubs and refuges	Waste water	Rice	Nurseries	Dirt bikes	Unknown	Total
Santa Barbara	809	735	3,004	278	4,308	1,708	319	218	1,354	1,005	46		35					13,822
Kern	2,232	643	141	1,557	3,477	1,081	546	671	24	993	23		9			35	4	11,437
Merced	34	143	126	4,440	329	2,537	432	739	687		87	1,335	76					10,964
San Luis Obispo	4,760	2,087	1,461	434	204	219	742	421	329	88	41							10,786
Monterey	960	588	5,041	5	895	108	790	417	13	1,501	4							10,322
Sonoma	2,500	945	3,208		122	738	42	541	44		1,559		18					9,716
Sacramento	1,750	4,303	2,226	42	642	141	106	427					16	20	35			9,707
Madera	5,535	221	688	5	44	2,117	497	340			11							9,457
Placer	2,432	5,818				5	13	357						85				8,709
El Dorado	5,740	2,144	132	27	4	184	62	113			5							8,411
Solano	1,715	3,736		36	66	1,214	35	1,096	75		10		86		191	27		8,288
Kings	23	4	433		20	246	4,767	455	369				1,197			31		7,544
Fresno	4,569	369		963	82	48	360	67	478		37							6,973
Tulare	1,906	455		1,504	106	1,037	299	688	387				16				41	6,438
Stanislaus	37	31	515	5,055	125	32	381	44		32	10							6,263
Contra Costa	460	4,212			283	64		972	24		25		55					6,096
Tehama	1,149	30		4,294	15	122	40	157	22		68		7					5,903
Alameda	370	2,662	909		291	30	11	1,251									38	5,563
Butte	2,857	1,039	272	249	109	182	177	307			95			121				5,408
San Joaquin	100	37	3,491	185	188	85	232	132	90		9				270			4,821
Santa Clara	1,110	2,211			126	203	148	177	317		105							4,398
Shasta	2,914	282			119	457	18	68			4							3,864
Nevada	2,379	133	65		584	302		4			105							3,572
Yuba	2,018	88	4	130	85	6	54	166			40	53		251				2,894
Napa	169	202	1,667		212	126	48	16			88							2,526
San Benito	436	300	106		455	55	68	175	522	187	90							2,393
Amador	629	119	669		406	18	5	230			15					20		2,110
Mariposa	1,875	56		47	10			8			29					38		2,064
Colusa	137	189	569	291	15	31	248	70	60		90			290				1,990
Sutter	100	30		558	17	109	172		9		92	616		45				1,748
Yolo		32			92		32	65	39		59	260	50					630
Glenn	257			9	120	92												477
Marin	29	112	21		7	5	33	48			46							302
**Total**	**51,990**	**33,958**	**24,749**	**20,111**	**13,558**	**13,300**	**10,678**	**10,441**	**4,841**	**3,807**	**2,791**	**2,264**	**1,564**	**812**	**496**	**151**	**84**	**195,594**
